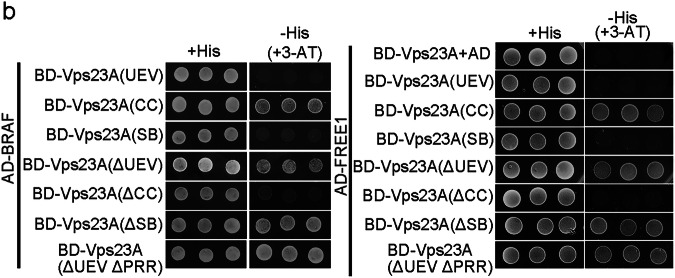# Author Correction: A plant Bro1 domain protein BRAF regulates multivesicular body biogenesis and membrane protein homeostasis

**DOI:** 10.1038/s41467-025-59866-0

**Published:** 2025-05-19

**Authors:** Jinbo Shen, Qiong Zhao, Xiangfeng Wang, Caiji Gao, Ying Zhu, Yonglun Zeng, Liwen Jiang

**Affiliations:** 1 Centre for Cell & Developmental Biology, State Key Laboratory of Agrobiotechnology, School of Life Sciences, The Chinese University of Hong Kong, Shatin, New Territories, Hong Kong China; 2 State Key Laboratory of Plant Physiology and Biochemistry, Department of Plant Sciences, College of Biological Sciences, China Agricultural University, Beijing, 100193 China; 3 Guangdong Provincial Key Laboratory of Biotechnology for Plant Development, School of Life Sciences, South China Normal University (SCNU), Guangzhou, 510631 China; 4 CUHK Shenzhen Research Institute, The Chinese University of Hong Kong, Shenzhen, 518057 China; 5 Present Address: State Key Laboratory of Subtropical Silviculture, Zhejiang A&F University, Linan, Hangzhou 311300 China

Correction to: *Nature Communications* 10.1038/s41467-018-05913-y, published online 17 September 2018

In the version of the article initially published, in Fig. 4b the image showing the interaction between BD-Vps23A(ΔUEV) and AD-FREE1 under -His (+3-AT) condition was unintentionally cropped from the image of the same pair under +His condition. The error has now been corrected with data from an independent repeat experiment and the corrected Fig 4b can be seen below. This correction does not affect any of the scientific findings and conclusions presented in the paper.

Original and corrected Fig. 4b.

Original Fig. 4b
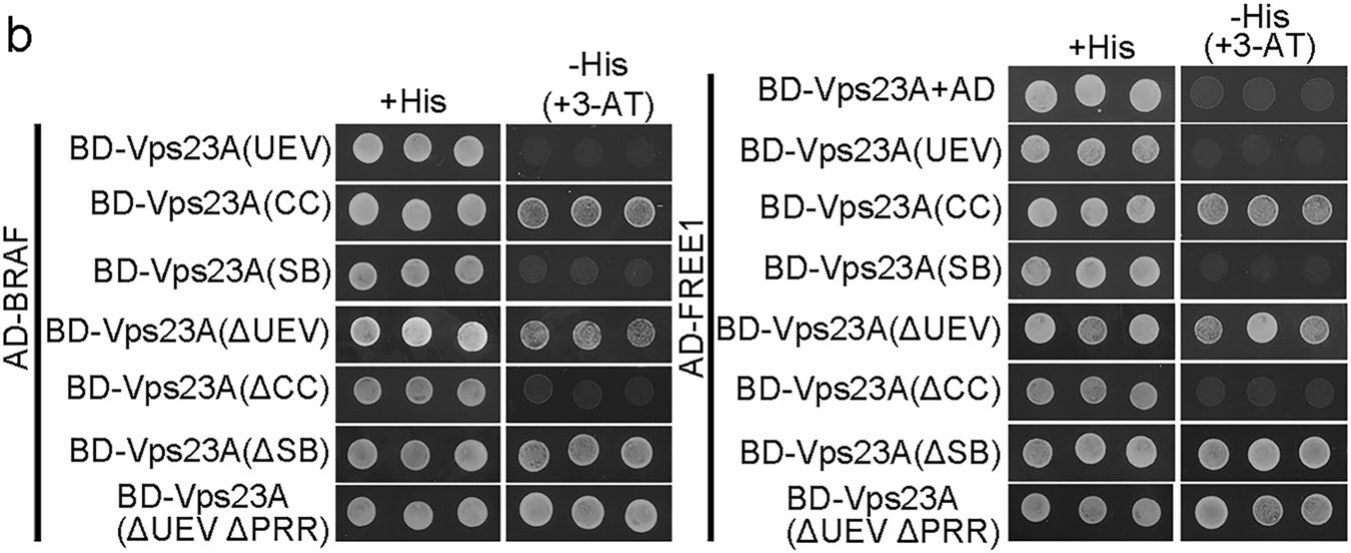


Corrected Fig. 4b