# Supplementary material for: A plant Bro1 domain protein BRAF regulates multivesicular body biogenesis and membrane protein homeostasis
Source: Nat Commun. 2018 Sep 17;9:3784. doi: 10.1038/s41467-018-05913-y (PMC6141507; doi:10.1038/s41467-018-05913-y)
Supplement: Supplementary file 1 — Supplementary Information [file 41467_2018_5913_MOESM1_ESM.pdf]

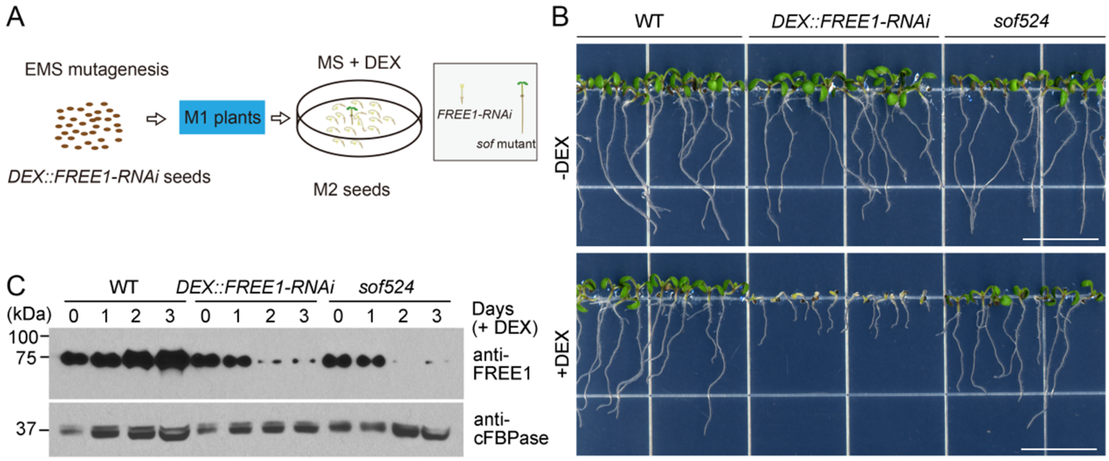

Supplementary Figure 1. Characterization of *sof524* mutant.

(A) Schematic illustration of *sof* screening procedures. Single insertion *DEX::FREE1-RNAi* seeds were EMS mutagenized, and surviving M2 seedlings with DEX induction were selected as putative *sof* mutants.

(B) Phenotypes of WT, *FREE1-RNAi*, and *sof524* seedlings grown vertically on MS agar plates with (+)/without (-) DEX for 7 days. Scale bar, 1 cm.

(C) FREE1 protein in *sof524* is reduced after DEX treatment. WT, *FREE1-RNAi*, and *sof524* seeds were grown on MS agar plates for 4 days, and the seedlings were then transferred to liquid MS medium containing 30 μM DEX. The DEX-treated five seedlings at each time point were subjected to protein extraction for immunoblot analysis with indicated antibodies. The cytoplasmic marker anti-cFBPase, a ubiquitously expressed cytosolic fructose-1,6-bisphosphatase, is used as a loading control.

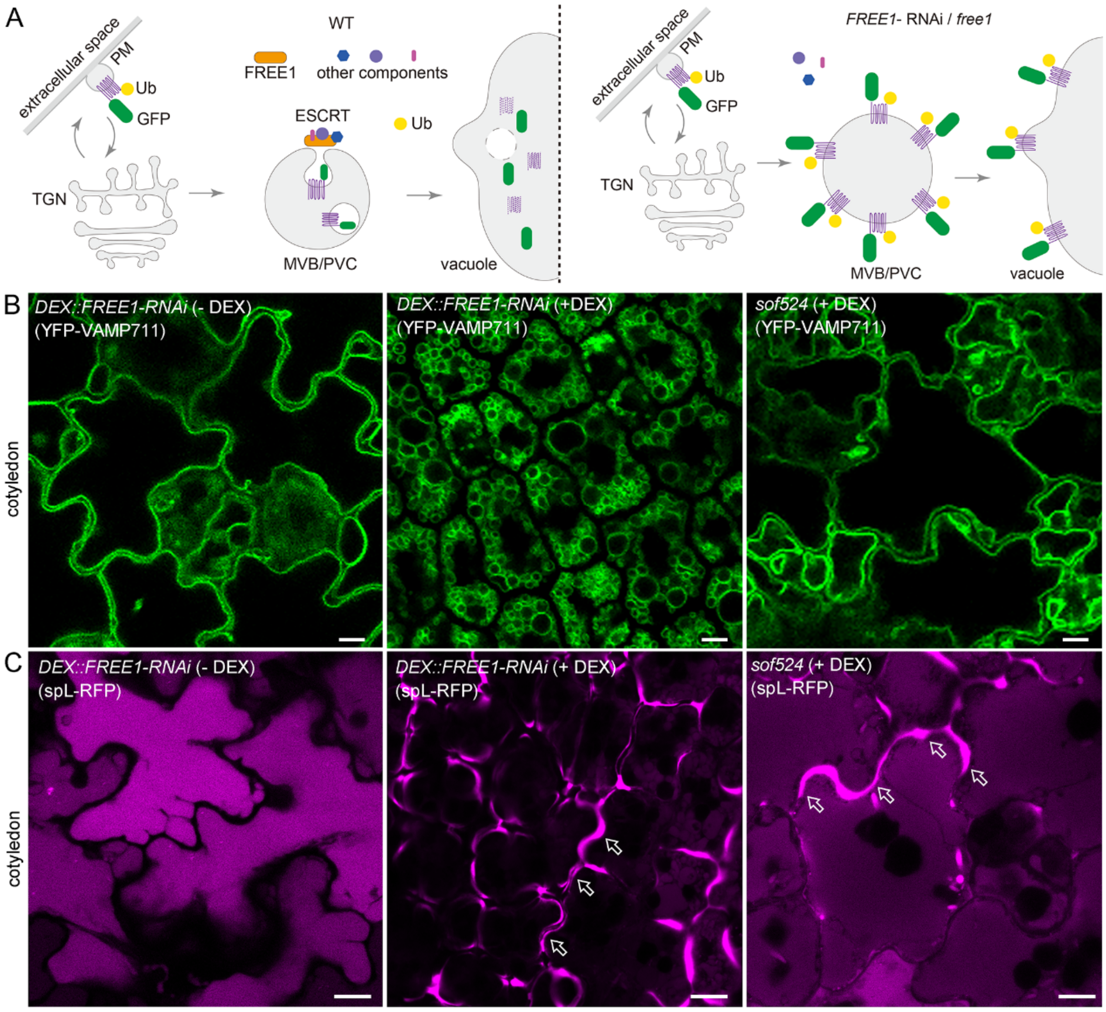

Supplementary Figure 2. *sof524* mutant convert the defect of *FREE1 RNAi* in vacuole morphology and vacuolar transport of soluble protein.

(A) Mode of FREE1 function in regulating vacuolar sorting of ubiquitinated membrane proteins. In plants, ubiquitinated plasma membrane proteins are internalized and subsequently delivered into endosomes. FREE1 protein, function together with other ESCRT components, then recognize ubiquitinated membrane proteins and sort them into intra-luminal vesicles (ILVs) inside the MVB/PVCs. ESCRT proteins also remove the ubiquitin (Ub) tag from the membrane proteins. When the MVB/PVCs fuse with vacuoles, the ILVs are released in the vacuolar lumen and membrane proteins are degraded. The depletion of FREE1 (*FREE1* RNAi or *free1* mutant) results in failure of ILVs formation and consequently causes accumulation of ubiquitinated membrane cargo in endosomes and the tonoplast.

(B) Vacuole morphology is converted in the *sof524* mutant. Confocal microscopic images of the cotyledon cells expressing the vacuolar membrane marker YFP-VAMP711 in indicated genotypes with (+)/without (-) DEX induction. Scale bar, 10 μm.

(C) Vacuolar transport of soluble protein marker spL-RFP is partially converted in the

sof524 mutant. Confocal microscopic images of cotyledon cells expressing spL-RFP in indicated genotypes with(+)/without(-) DEX induction. The arrows indicate the secretion of spL-RFP. Scale bar, 10 µm.

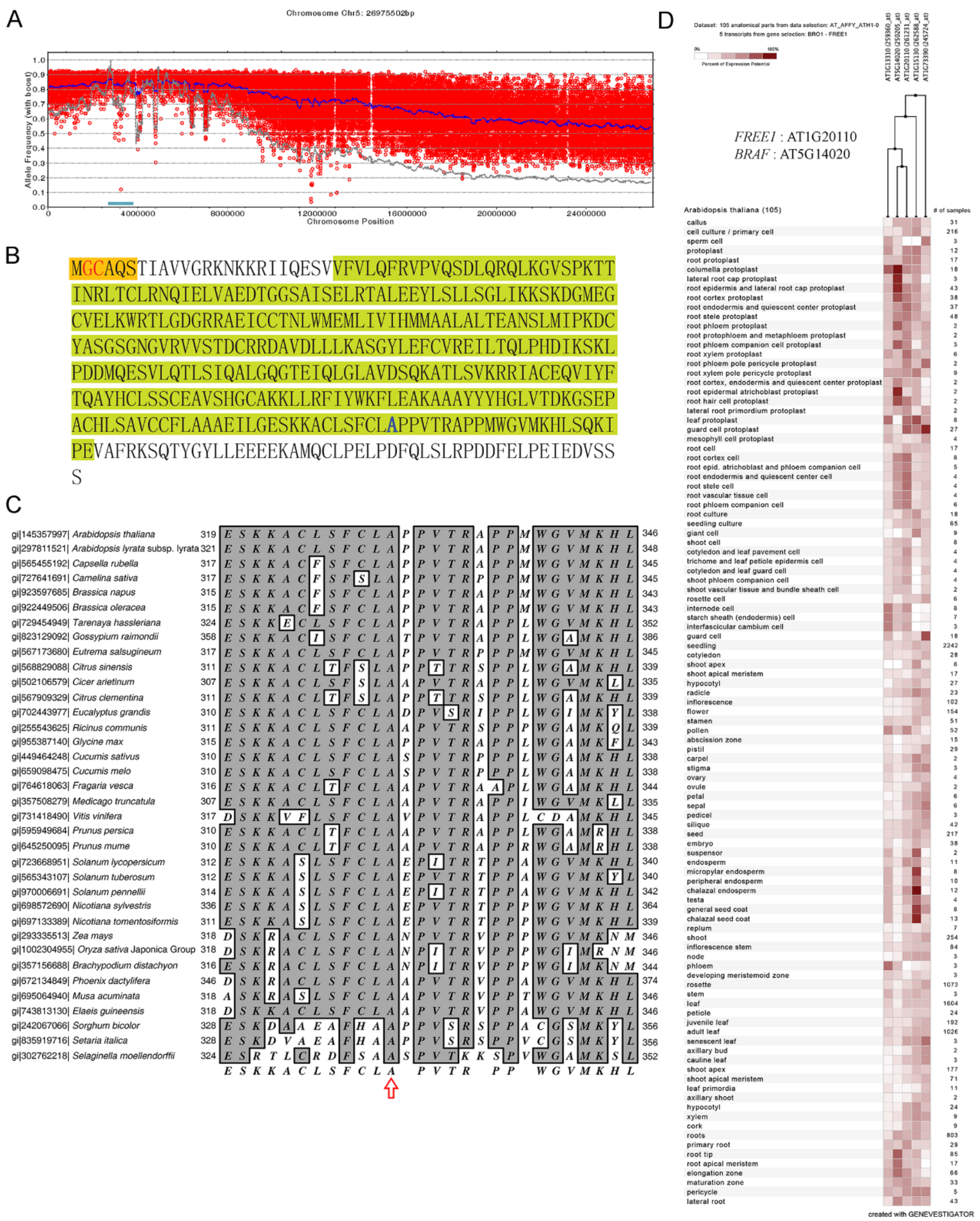

Supplementary Figure 3. Next-Generation Sequencing of *sof524*, and sequence analysis of BRAF proteins in plants.

(A) Allele frequency (AF) analysis result of *sof524* on chromosome 5. One specific Col-allele peak appears in the left region as highlighted by the light blue bar, which is predicted as mapping interval. Red circle dots indicate allele frequency estimations on individual markers. The blue line shows the average allele frequency estimations within 200 kb windows with a 5 kb step size. The dashed line in grey indicates window-based boot value of allele frequency (= summation-of-single-marker-AF divided by number-of-markers with minimum

quality score involved).

(B) Protein sequence of BRAF. The green parts highlight Bro1-domain and the potential N-myristoylation and S-acylation in the second and third amino-acids are highlighted in red. The alanine mutation in *sof524* is highlighted in blue.

(C) Alignment of the plant BRAF homologs showing conservation of alanine residue identified in *sof524* mutation (arrow).

(D) Hierarchical clustering of tissue-specific expression patterns of Arabidopsis Bro1-domain genes and *FREE1* by Genevestigator database. Color bar at top indicates percent of expression potential. The hierarchical clustering was calculated by Euclidean distance. Note the nearest cluster between *BRAF* and *FREE1*.

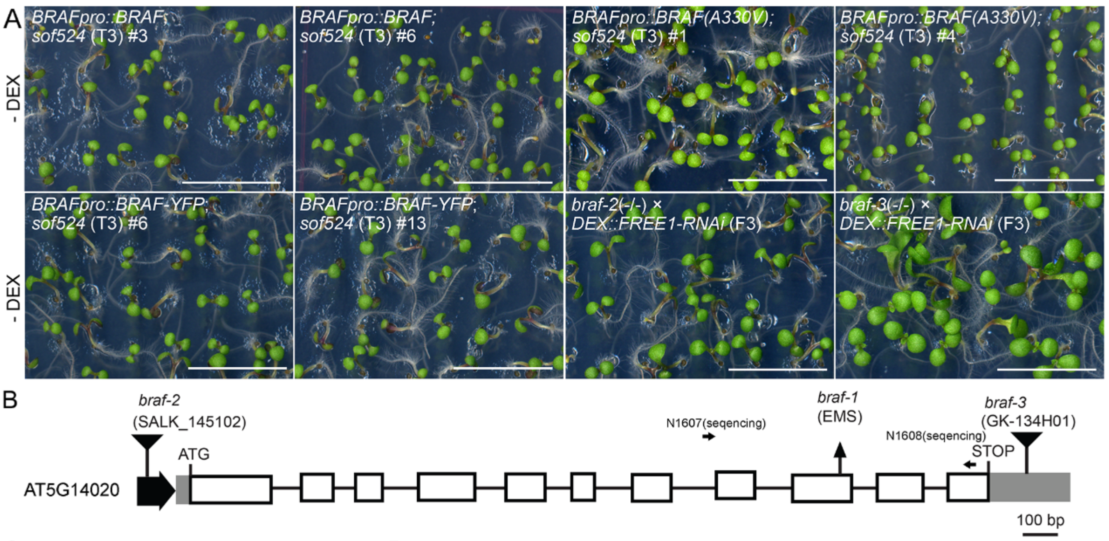

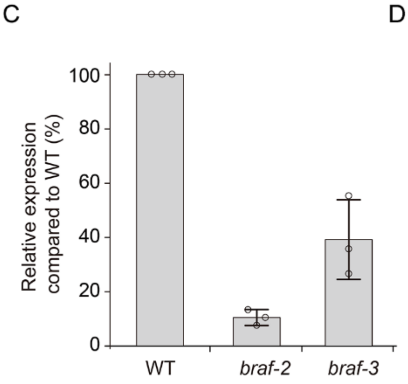

D

Genotype of parental line		Phenotype of progeny: MS Survival	MS Lethal	MS Hyg R^-	MS +Hyg +DEX Survival	MS +Hyg +DEX Lethal	MS +Hyg +DEX Hyg R^-
WT (Columbia-0)		215	0	-	0	0	317
DEX::AtBRO1-RNAi x *braf-2*(-/-) (F1)		258	0	-	0	259	64
DEX::SH3P2-RNAi x *braf-2*(-/-) (F1)		244	1	-	0	153	61
DEX::SNF7.1(L32W) *braf-2*(-/-) (T1)	#9	37	0	-	0	57	3
	#10	43	0	-	0	68	6
	#16	59	0	-	0	80	11
DEX::Vps4(E232Q) *braf-2*(-/-) (T1)	#1	32	0	-	0	75	2
	#8	53	0	-	0	87	8
	#13	73	0	-	0	88	12

Supplementary Figure 4. Complementation analysis and characterization of BRAF T-DNA insertion mutants.

(A) The phenotypes of *sof524* seedling expressing indicated fusion proteins or double homozygous mutant growth on MS plates without (-) DEX. Scale bar, 1 cm.

(B) A diagram of *BRAF* genomic region showing the position of the EMS-induced G-to-A mutation in *sof524* plants and that of T-DNA insertions in *braf*-2 and *braf*-3 mutants.

(C) The qRT-PCR assays of *BRAF* transcripts in different T-DNA insertion mutants using P1 (O1232)/P2 (O189) primers, respectively.

(D) Phenotype analysis of indicated progeny. The phenotypes of 7-d-old seedling of indicated genotypes grown on MS plates or supplied with (+) DEX and hygromycin (Hyg) were analyzed. Note no seedling survived when grown on MS plate with Hgy and DEX, indicating the *braf-2* mutant does not convert the seedling lethal phenotype of ESCRT-related component.

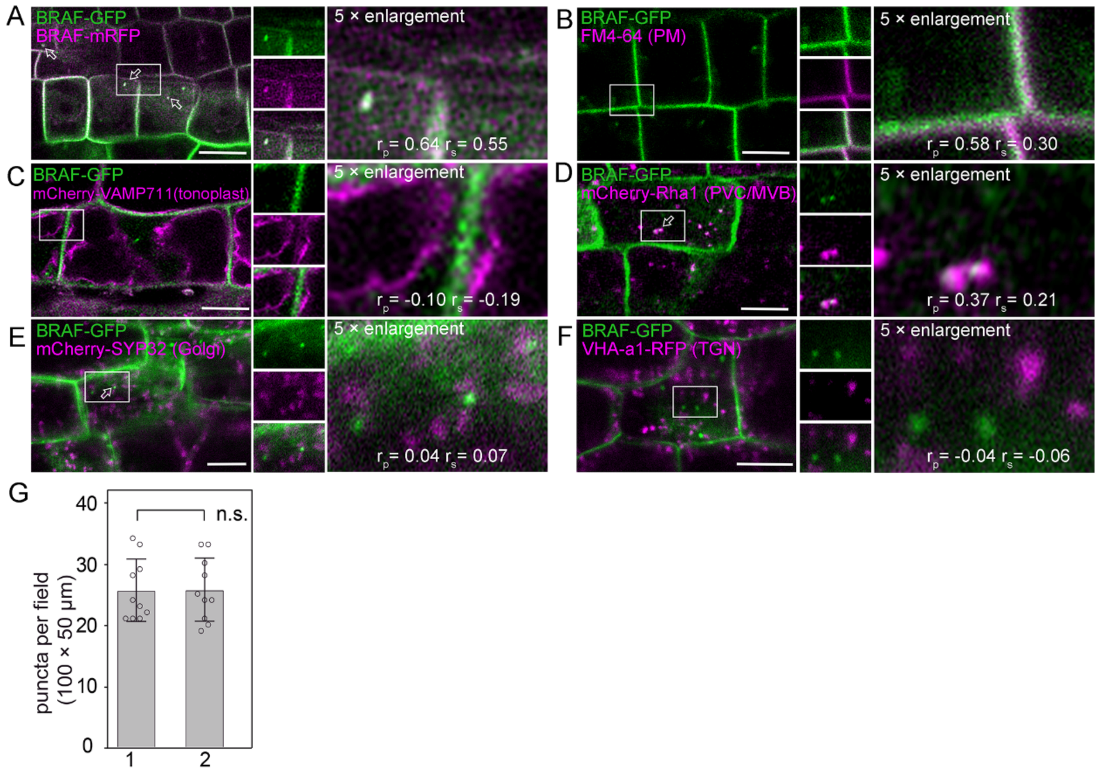

Supplementary Figure 5. Subcellular localization of BRAF.

(A-F) Confocal colocalization analysis of BRAF-GFP with the BRAF-mRFP (A), PM marker FM4-64 (B), the tonoplast marker mCherry-VAMP711 (C), the MVB/PVC marker mCherry-Rha1 (D), the Golgi marker mCherry-SYP32 (E), or the TGN/EE marker VHA-a1-RFP (F) in root epidermal cells of 5-d-old Arabidopsis seedlings. Separated images of each channel in the white outline area are shown in the middle (from top to bottom: GFP, RFP, merged). The regions within the white outline are enlarged in the right panels (magnification: 5×). Colocalization relationship was calculated by Pearson–Spearman correlation. Scale bar, 10 μm.

(G) The numbers of intracellular punctae per root section of *UBQPro::BRAF-GFP* (column 1) or *BRAFPro::BRAF-YFP* (column 2) are quantified. The results were obtained from 10 individual seedlings. n.s., P>0.05 in Student's *t*-test.

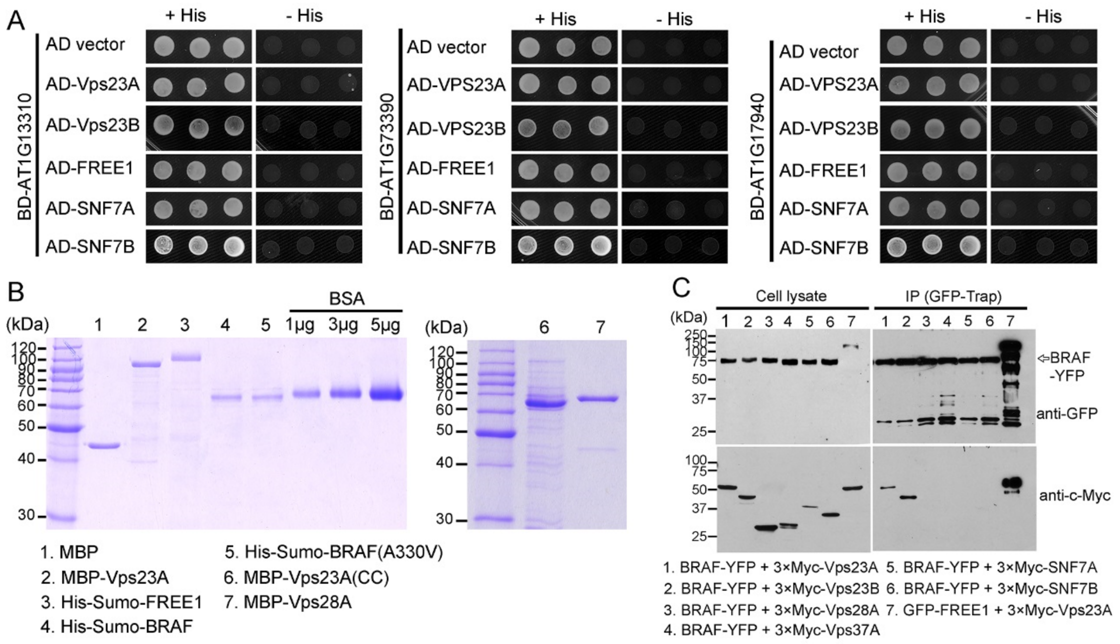

Supplementary Figure 6. Mapping interactions between Arabidopsis Bro1-domain proteins and ESCRT components.

(A) Y2H analysis of the binary interactions of Arabidopsis Bro1-domain proteins AT1G13310, AT1G73390, or AT1G17940 with ESCRT components. Transformed yeast cells were grown on either synthetic complete medium lacking leucine and tryptophan (with histidine, +His) as a transformation control, or synthetic complete medium lacking leucine, tryptophan, and histidine (without histidine, -His) for interaction assays.

(B) Purified recombinant proteins which were subjected to *in vitro* binding assay.

(C) Immunoprecipitation (IP) assay shows association between BRAF and Vps23, but not ESCRT-I component Vps28A, Vps37A nor ESCRT-III component SNF7A, SNF7B. Arabidopsis protoplasts expressing BRAF-GFP with Myc-tagged ESCRT components were subjected to protein extraction and IP with GFP-trap followed by immunoblot with indicated antibodies. The GFP-FREE1 and 3×Myc-Vps23A pair is used as a positive control.

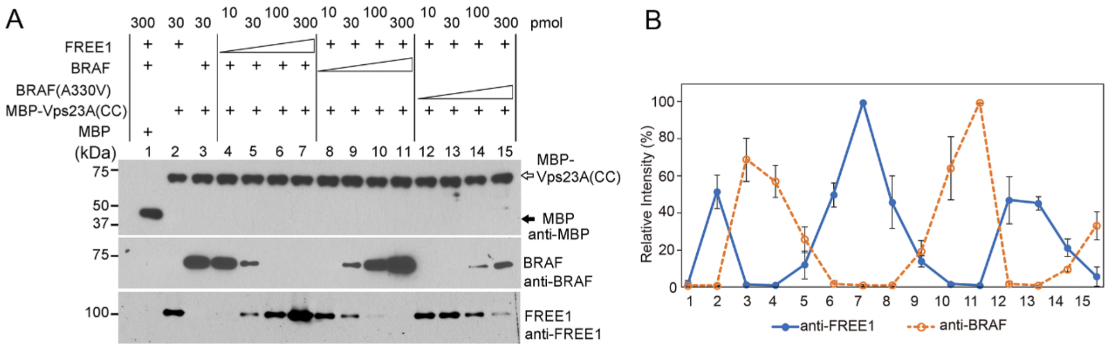

Supplementary Figure 7. BRAF competes with FREE1 for Vps23A coiled-coil domains binding.

(A) Binding assay of MBP-Vps23A(CC) with 30 pmol of BRAF was performed in the presence of 10, 30, 100, or 300 pmol of FREE1 as indicated. Note that BRAF binding becomes weaker in the presence of an excess amount of FREE1. Binding assay of MBP-Vps23A(CC) with constant FREE1 was also performed using increased amounts of BRAF or BRAF(A330V) as indicated. Anti-MBP, anti-BRAF, and anti-FREE1 antibodies were used to detect beads-retained materials.

(B) The pull-down efficiency. The amount of proteins on the sepharose was compared with that of the control pair (as 100% binding affinity) of MBP-Vps23A(CC)/FREE1 (lane 7) or MBP-Vps23A(CC)/BRAF (lane 11). The intensity of the pull-down was quantified using IMAGEJ software and normalized by the MBP-Vps23A(CC) intensity. Error bars are the S.D. from three independent experiments.

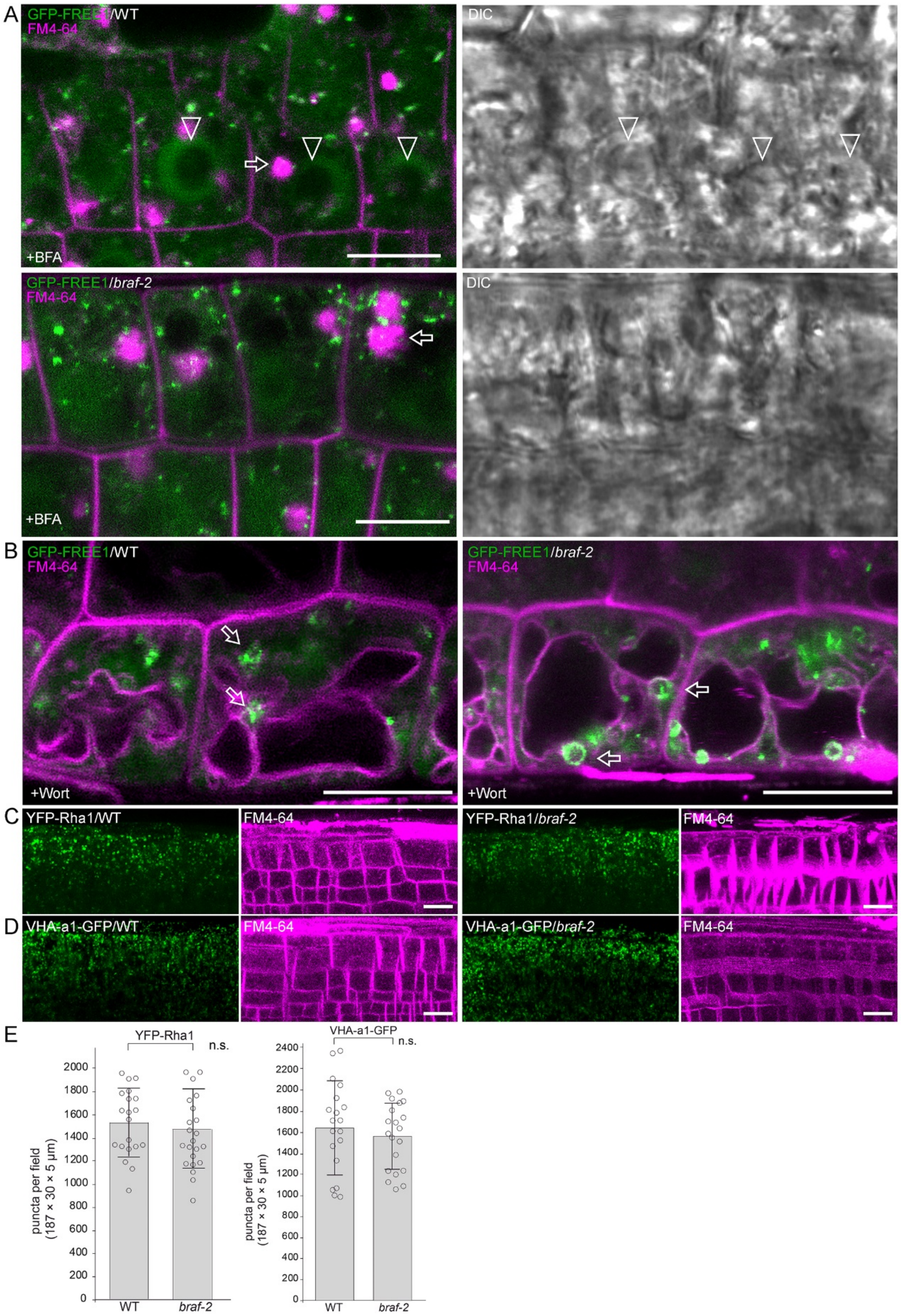

Supplementary Figure 8. GFP-FREE1 punctae in WT and *braf-2* mutant plants are on MVB/PVCs.

(A) WT and *braf-2* mutant plants expressing the GFP-FREE1 were subjected to FM4-64 uptake for 5 min and BFA treatment for 2 h, followed by confocal

analysis in root cells. Arrows indicate the BFA bodies. Note that some of the GFP-FREE1 signals are also presented in the nucleus, in which the arrow heads indicate the nucleolus.

(B) WT and *braf-2* mutant plants expressing the GFP-FREE1 were incubated with FM4-64 for 6 hrs and subjected to wortmannin (Wort) treatment for 40 min, followed by confocal imaging in root cells. Arrows indicate the localization of FREE1 to the surface of the enlarged MVB/PVCs that appeared as ring-like structures after wortmannin treatment. Scale bars, 10 μm.

(C-E) Confocal images of YFP-Rha1 (C) or VHA-a1-GFP (D) punctae in *braf*-2 mutant and WT root cells. Confocal images were collected from the root epidermal cells of the basal meristem region. Analysis of the number of punctae per root section by Z stack projection is quantified (E). Ten slices were collected in a total thickness of 5 μm for generating the 3D projection image. The results were obtained from 10 individual seedlings. Error bars represent the S.D. n.s., $P > 0.05$ in Student's *t*-test. FM4-64 was used to visualize the cell plasma membranes. Scale bars, 10 μm.

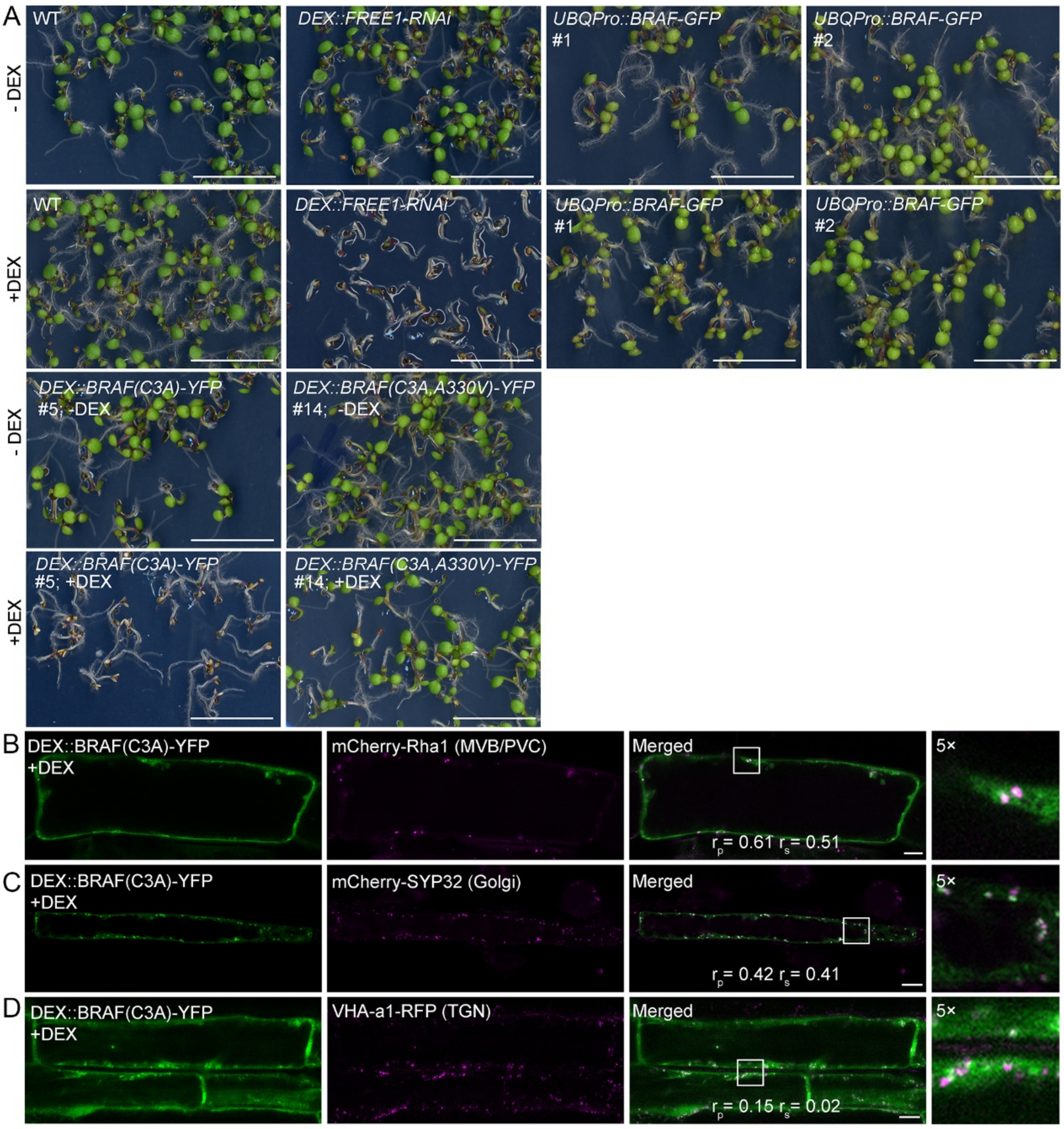

Supplementary Figure 9. Characterization of BRAF-overexpression plants and BRAF(C3A)-overexpression mutants.

(A) The phenotype of 7-d-old seedlings of indicated genotypes growth on MS plates with (+) or without (-) DEX. Scale bar, 1 cm.

(B-D) Partially colocalization of BRAF(C3A)-YFP with the MVB/PVC marker mCherry-Rha1 and Golgi marker mCherry-SYP32 in Arabidopsis root epidermal cells of the elongation region. Seedlings expressing DEX::BRAF(C3A)-YFP were crossed with MVB/PVC marker mCherry-Rha1 (B), Golgi marker mCherry-SYP32 (C), or TGN marker VHA-a1-RFP (D) and colocalization was examined under a confocal microscope after 30 μM DEX for 24 h induction of 6-d-old transgenic Arabidopsis seedlings. Pearson (r_p) and Spearman (r_s) correlation coefficients were calculated from 10 individual plants. Scale bar, 10 μm.

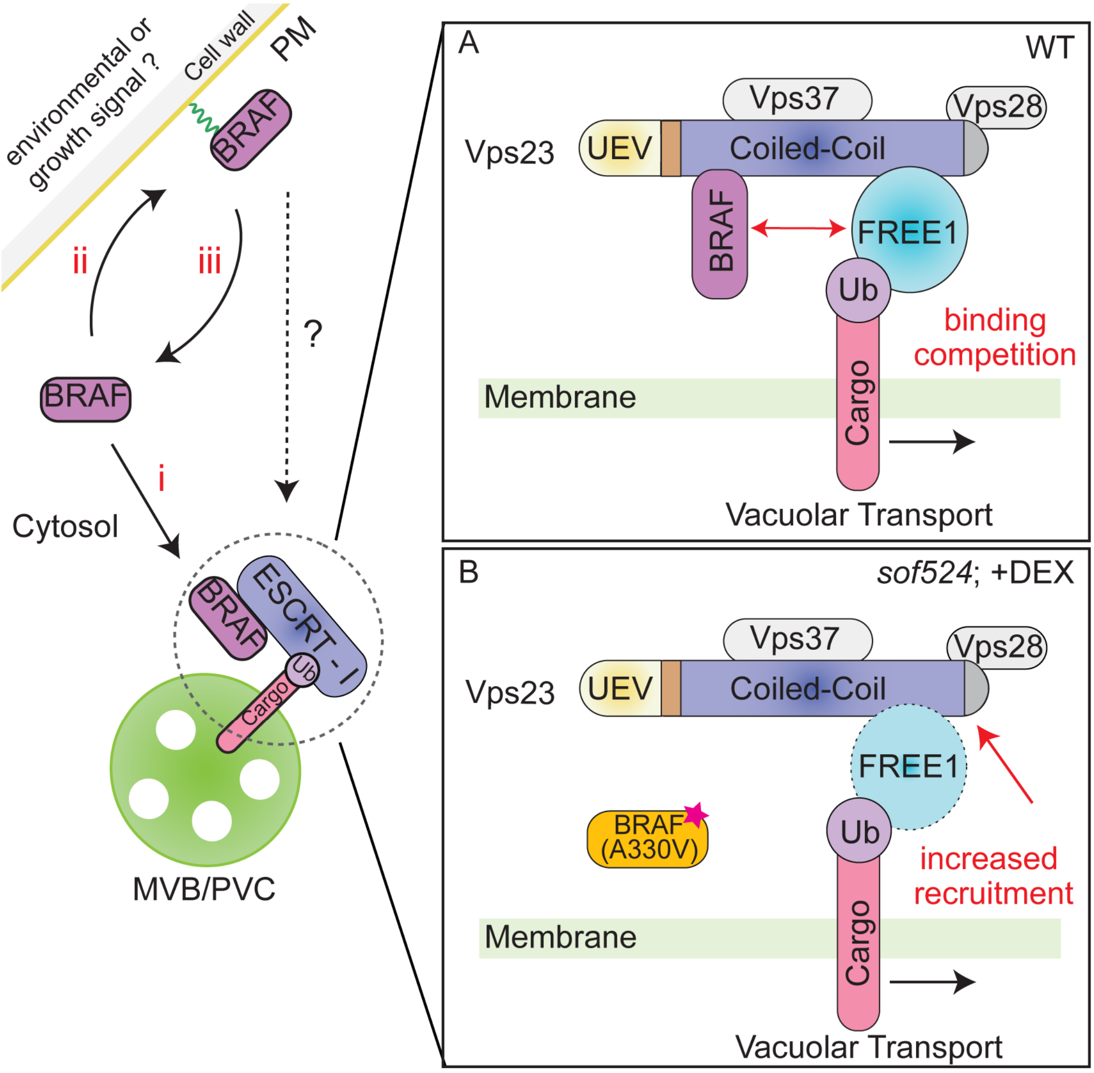

Supplementary Figure 10. Working model of BRAF function at the MVB/PVCs membrane and PM localization of the S-acylated BRAF.

BRAFs are cytoplasmic pool proteins that can be recruited to (i) MVB/PVCs via the ESCRT components or (ii) The plasma membrane (PM) upon their S-acylation in WT plant. (iii) The PM-localized S-acylated BRAF proteins can be released from the PM into the cytoplasm upon their de-acylation, likely resulting in increased cytosolic pool of BRAFs for MVB/PVCs recruitment. It remains unknown if the PM-localized S-acylated BRAFs could be recruited directly to MVB/PVCs when released from the PM upon their de-acylation (dash line with question mark).

(A) In WT plants, BRAFs are recruited to the MVB/PVCs membrane, and competed with FREE1 through direct binding to the ESCRT-I component Vps23, thus negatively regulating FREE1 function at MVB/PVCs.

(B) The *sof524* contains the alanine to valine mutation of BRAF in the DEX::*FREE1-RNAi* plants. BRAF(A330V) mutation has a lower binding ability to Vps23, compared to the WT version. In the DEX-treated *sof524* plants, the decreased

competition ability of BRAF(A330V) leads to the increased recruitment of the RNAi-decreased FREE1 to MVB/PVCs, thus complementing the defects in *FREE1-RNAi* plant.

Thus, the plant unique BRAF functions as a FREE1 negative regulator of the ESCRT pathway in regulating MVB/PVCs function and membrane protein homeostasis.

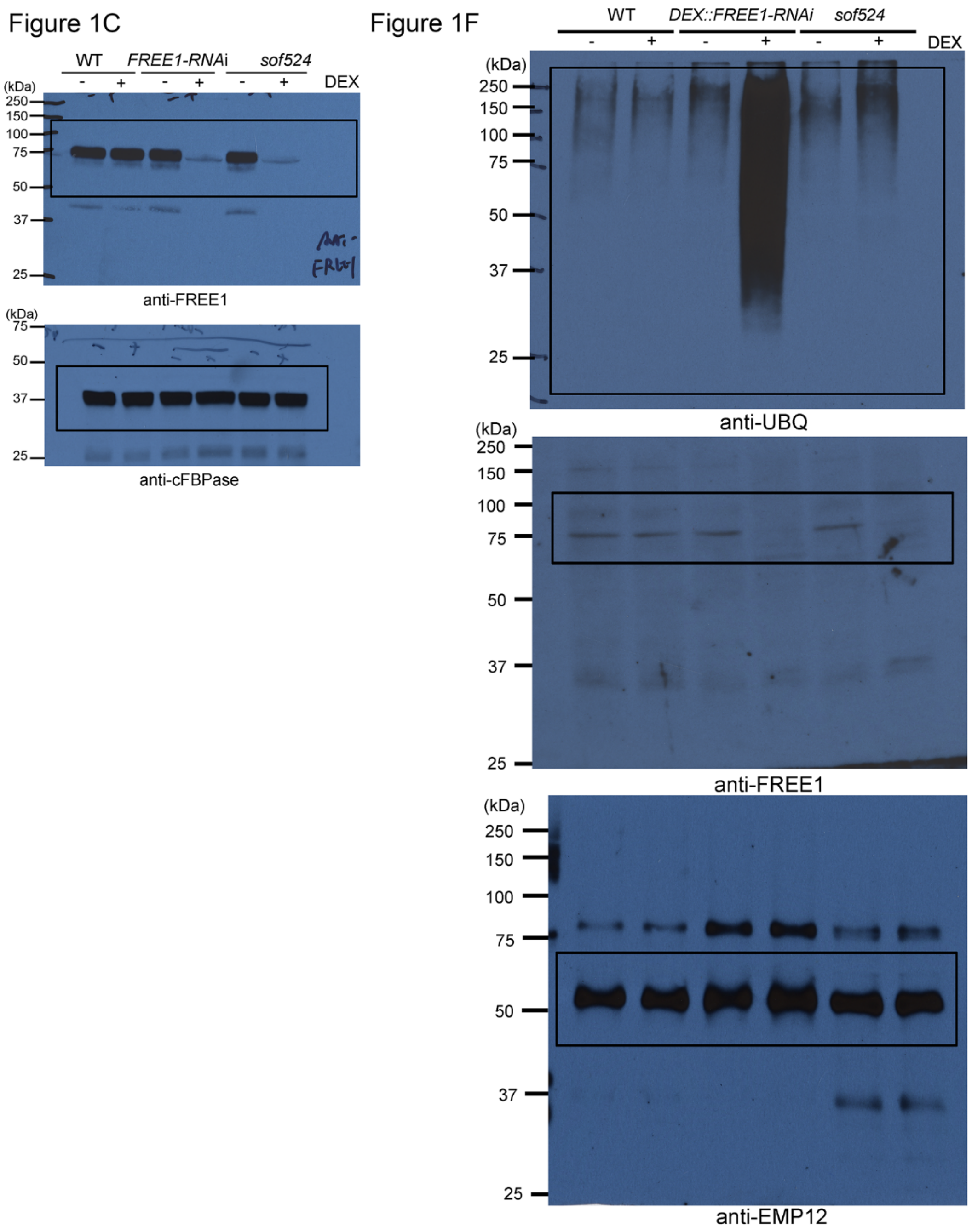

Supplementary Figure 11. Uncropped images of blots shown in Fig. 1.

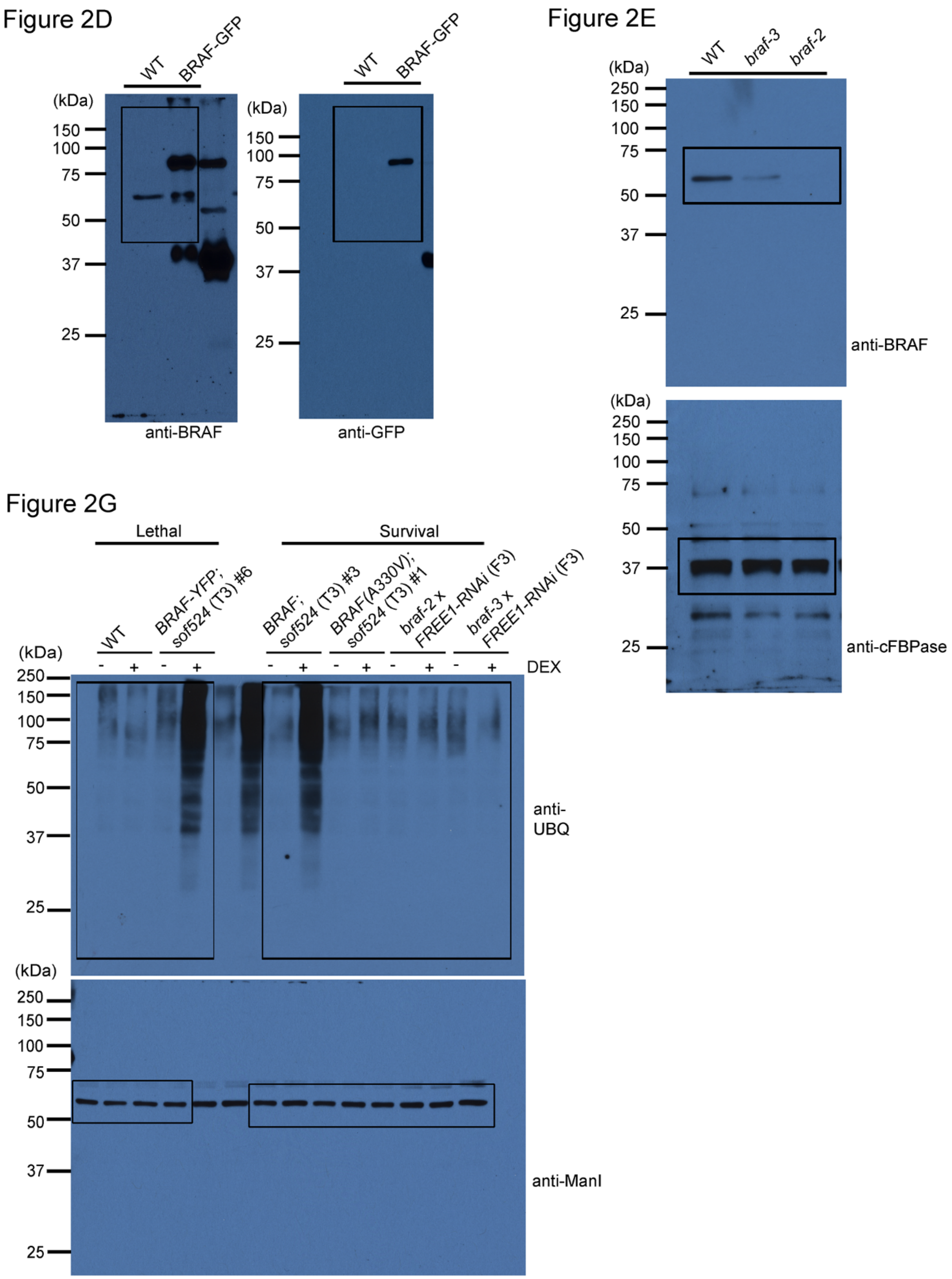

Supplementary Figure 12. Uncropped images of blots shown in Fig. 2.

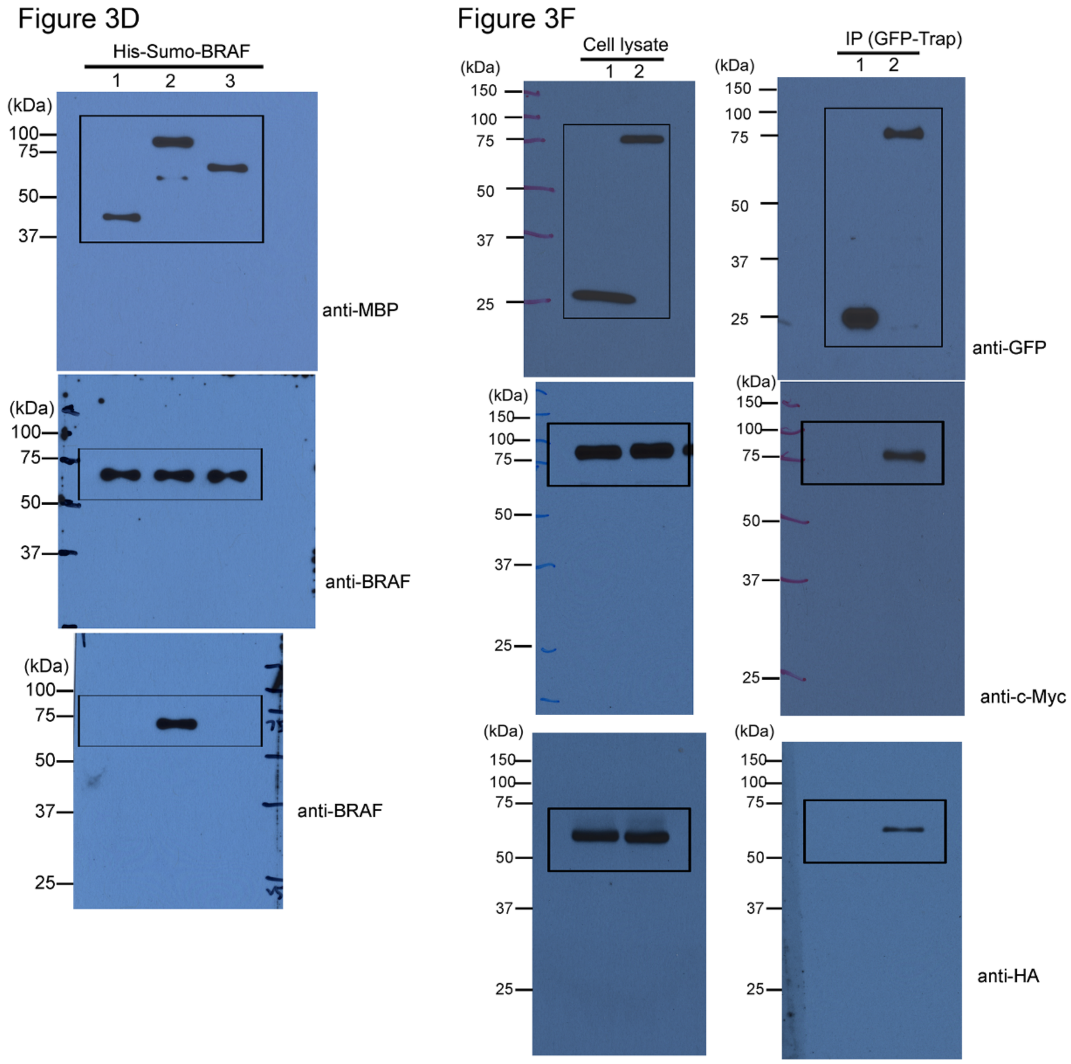

Supplementary Figure 13. Uncropped images of blots shown in Fig. 3.

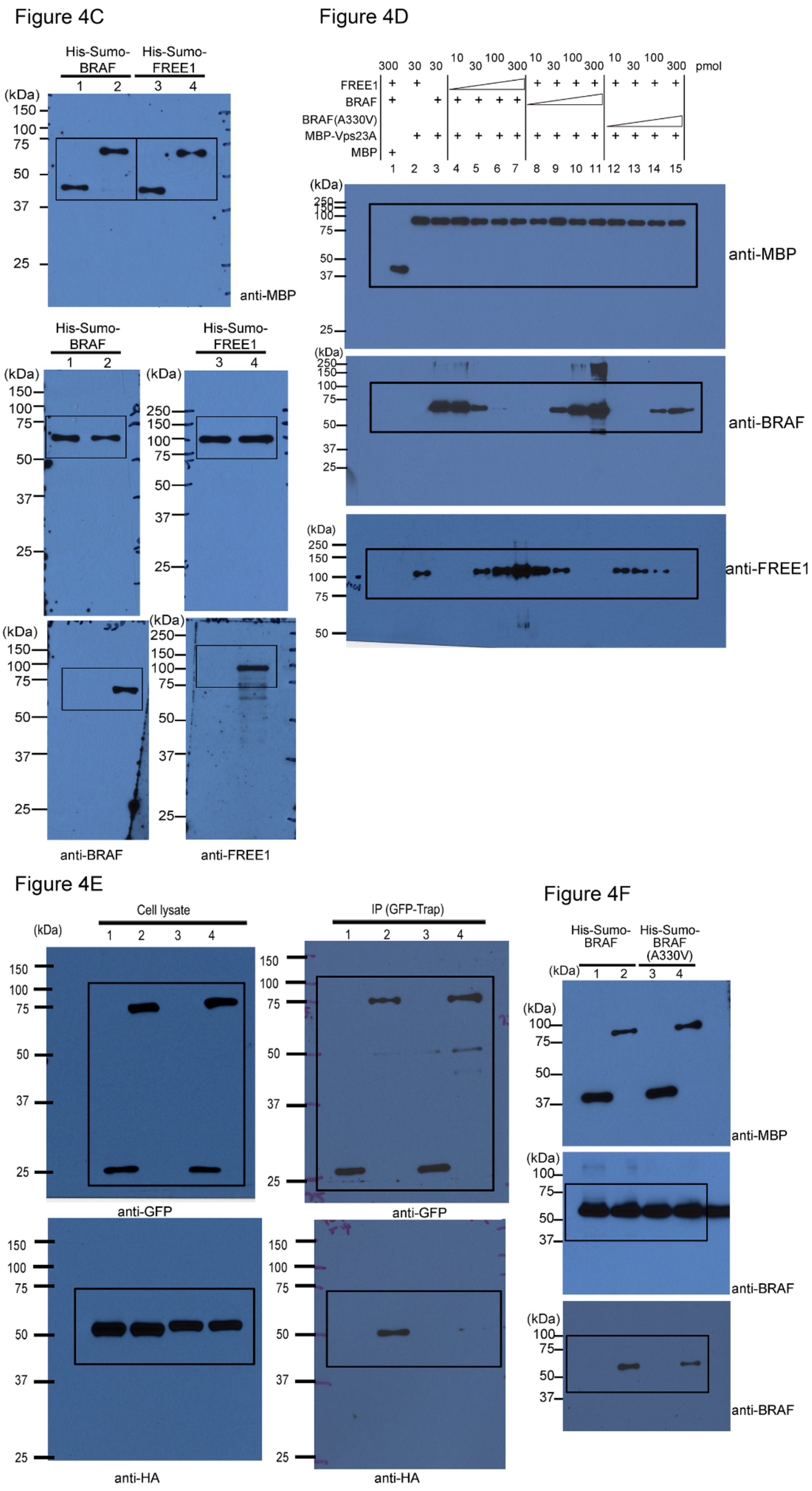

Supplementary Figure 14. Uncropped images of blots shown in Fig. 4.

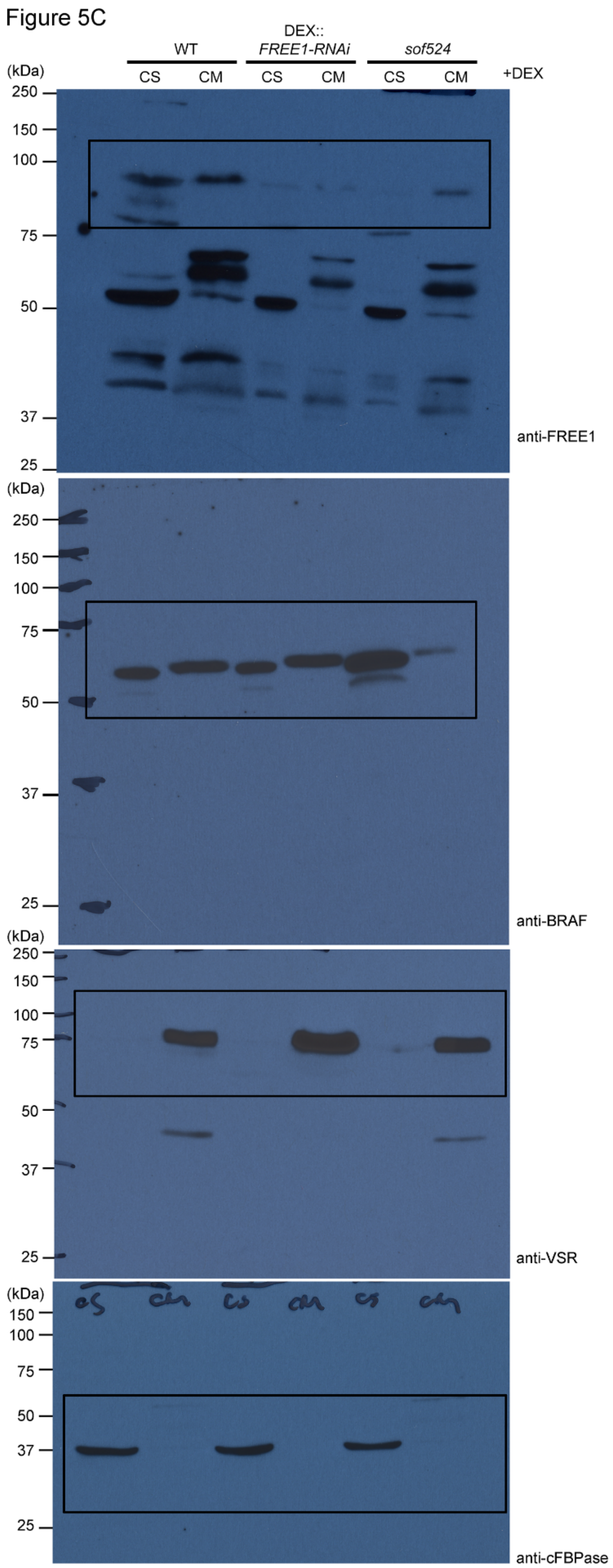

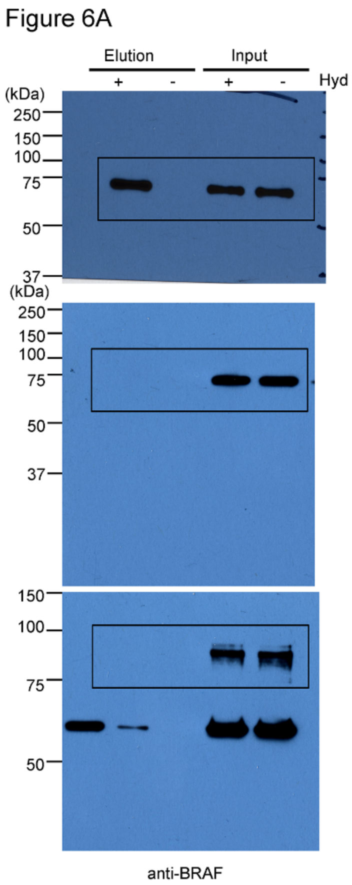

Supplementary Figure 15. Uncropped images of blots shown in Fig. 5 and Fig. 6.

Figure 7A

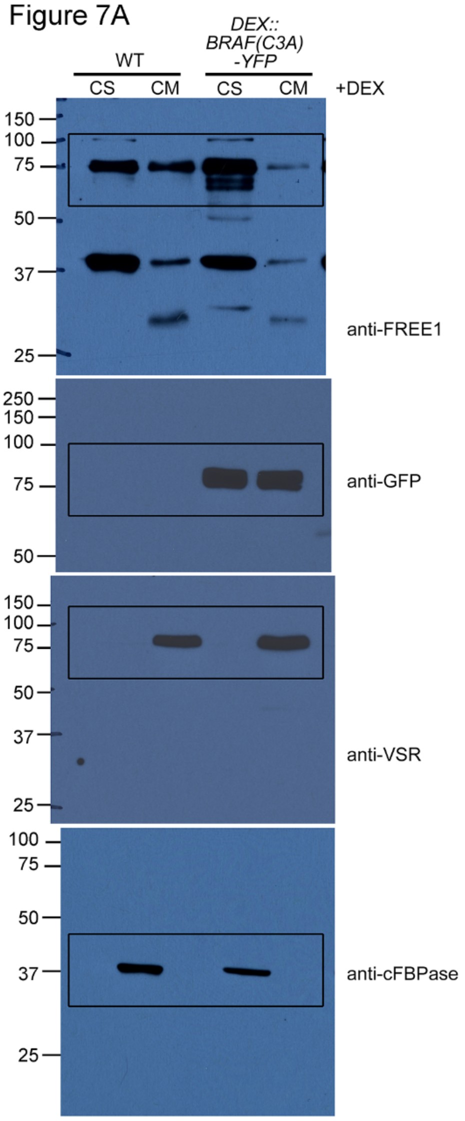

Figure 7C

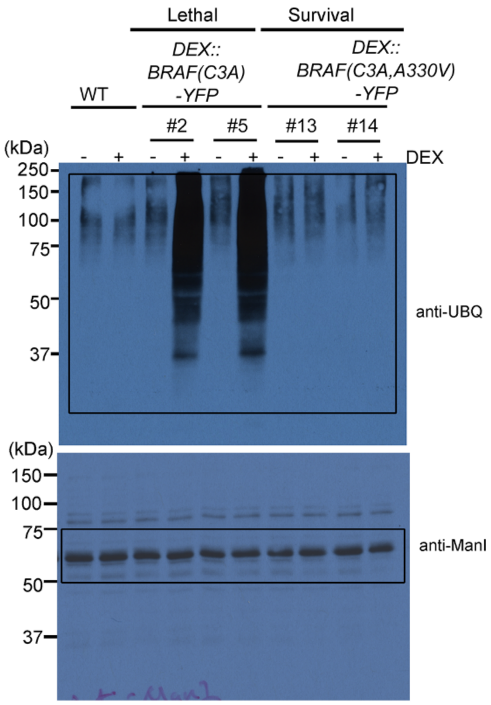

Supplementary Figure 16. Uncropped images of blots shown in Fig. 7.

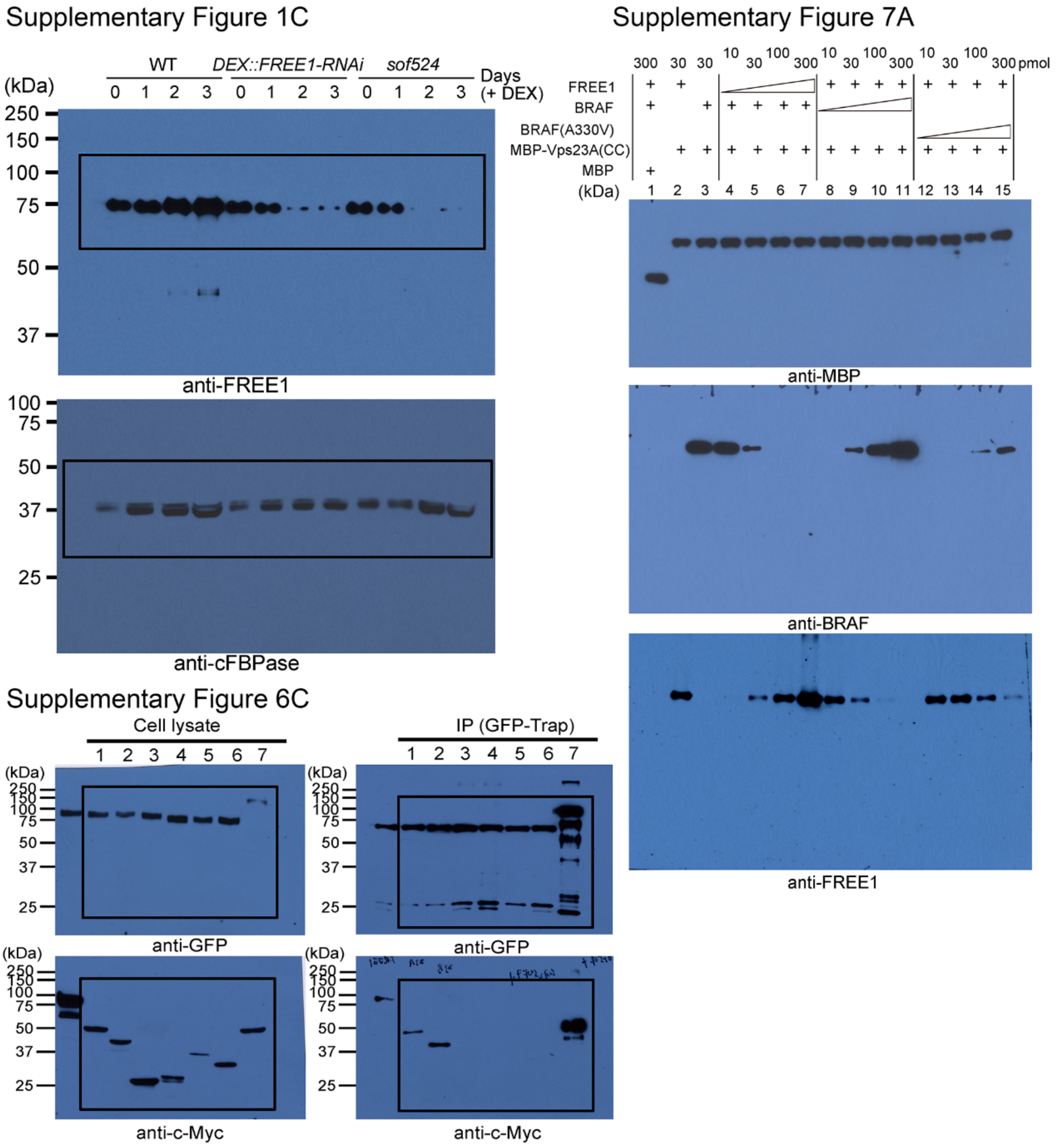

Supplementary Figure 17. Uncropped images of blots shown in Supplementary Figures.

Supplementary Table 1. Primers used in this study.

Construct name	Primer name and sequence (from 5' start to 3' end)
pDONR-BRAF/ pDONR-BRAF(A330V)	N1617-GW-F GGGGACAAGTTTGTACAAAAAAGCAGGCTTG ATG GGG TGT GCT CAA TCT ACT ATT GCT GTT GTG GGA<br>N1618-GW-R GGGGACCACTTTGTACAAGAAAGCTGGGTA TGA GGA AGA GAC ATC CTC TAT TTC AGG CAG TTC AAA
His-SUMO-BRAF	O304-BamHI-F GGG GGA TCC ATG GGG TGT GCT CAA TCT ACT ATT GCT GTT GTG GGA<br>O305-HindIII-R GGG AAG CTT TCA TGA GGA AGA GAC ATC CTC TAT TTC AGG CAG TTC AAA
MBP-Vps23A	O1742-BamHI-F GGG GGA TCC ATG GTT CCC CCG CCG TCT AAT CCG CAG CAG GTT<br>O1743-SalI-R GGG GTC GAC TCA TGA ATG TAA CCT ACC TGC GAT GGC TGC AAC CTG AA
pGBKT7-BRAF/ pGADT7-BRAF	N1721-EcoRI-F CCG GAA TTC ATG GGG TGT GCT CAA TCT ACT ATT GCT GTT GTG GGA<br>N1722-BamHI-R GGG GGA TCC TCA TGA GGA AGA GAC ATC CTC TAT TTC AGG CAG TTC AAA
pBI221-BRAF-5×HA/ pBI221-BRAF-YFP	O188-SpeI-F GGG ACT AGT ATG GGG TGT GCT CAA TCT ACT ATT GCT GTT GTG GGA<br>O1766-XhoI-R GGG CTC GAG TGA GGA AGA GAC ATC CTC TAT TTC AGG CAG TTC AAA
pBI221-3×HA-BRAF	O188-SpeI-F GGG ACT AGT ATG GGG TGT GCT CAA TCT ACT ATT GCT GTT GTG GGA<br>O892-KpnI-R GGG GGT ACC TCA TGA GGA AGA GAC ATC CTC TAT TTC AGG CAG TTC AAA
pDONR-BRAFpro:: BRAF-YFP	O502-GW-pro-F GGGGACAAGTTTGTACAAAAAAGCAGGCTTG TAA TCA CCA CCA CCA CCA CCC ATT<br>O1768-Bridge-R CAC AAC AGC AAT AGT AGA TTG AGC ACA CCC CAT GCT CTC TTT CTC TCT CTC TCT CTC TCT<br>O1767-Bridge-F AGA GAG AGA GAG AGA GAG AAA GAG AGC ATG GGG TGT GCT CAA TCT ACT ATT GCT GTT GTG<br>O1769-GW-YFP-R GGGGACCACTTTGTACAAGAAAGCTGGGTA TTA TTC TTT GTA TTG TTC ATC CAT GCC ATG TGT AAT CCC
pDONR-BRAFpro::	O502-GW-pro-F

BRAF<br>pDONR-BRAFpro::<br>BRAF(A330V)	O1768-Bridge-R<br>O1767-Bridge-F<br>N1619-GW-R GGGGACCACTTTGTACAAGAAAGCTGGGTA TCA TGA GGA AGA GAC ATC CTC TAT TTC AGG CAG TTC AAA
pGBKT7<br>BD-Vps23A-UEV	P1428-Vps23A(UEV)-EcoRI-F CCG GAA TTC ATG TCC AAC AAG TGG TTG ATC CGG CAA CAT CTA<br>P1429-Vps23A(UEV)-BamHI-R GGG GGA TCC TCA TCG AGA ATA AAG AGG CGG ATC ACG AGC AAA AGC
pGBKT7<br>BD-Vps23A-CC	P1430-Vps23A(CC)-EcoRI-F CCG GAA TTC ATG TTT GCT CGT GAT CCG CCT CTT TAT TCT CGA CG<br>P1431-Vps23A(CC)-BamHI-R GGG GGA TCC TCA ACA TTC AAA AGC ATT ATC CAC ATC CAA ATC AAC
pGBKT7<br>BD-Vps23A-SB	P1432-Vps23A(SB)-EcoRI-F CCG GAA TTC ATG GAT TTG GAT GTG GAT AAT GCT TTT GAA TGT GG<br>P1433-Vps23A(SB)-BamHI-R GGG GGA TCC TCA AGA ACC CGT GGC TCG GTG GAA GAA CTG TTC TCT
pGBKT7<br>BD-Vps23A-ΔUEV	P1434-Vps23A(ΔUEV)-EcoRI-F CCG GAA TTC ATG TTT GCT CGT GAT CCG CCT CTT TAT TCT CGA CG<br>P1427-Vps23A-BamHI-R GGG GGA TCC TCA TGA ATG TAA CCT ACC TGC GAT GGC TGC AA
pGBKT7<br>BD-Vps23A-ΔCC	P1435-Vps23A(ΔCC)-Linker-F GCT GCT TTT GCT CGT GAT CCG CCT CTT TAT TCT CGA GAT TTG GAT GTG GAT AAT GCT TTT GAA TGT GGT GAC ACA<br>P1427-Vps23A-BamHI-R GGG GGA TCC TCA TGA ATG TAA CCT ACC TGC GAT GGC TGC AA<br>P1426-Vps23A-EcoRI-F CCG GAA TTC ATG GTT CCC CCG CCG TCT AAT CCG CAG CAG<br>P1436-Vps23A(ΔCC)-Linker-R TGT GTC ACC ACA TTC AAA AGC ATT ATC CAC ATC CAA ATC TCG AGA ATA AAG AGG CGG ATC ACG AGC AAA AGC AGC
pGBKT7<br>BD-Vps23A-ΔSB	P1426-Vps23A-EcoRI-F CCG GAA TTC ATG GTT CCC CCG CCG TCT AAT CCG CAG CAG<br>P1431-Vps23A(CC)-BamHI-R GGG GGA TCC TCA ACA TTC AAA AGC ATT ATC CAC ATC CAA ATC AAC
pGBKT7<br>BD-Vps23A(ΔUEV ΔPRR)	P1803-Vps23A(ΔUEVΔPRR)-EcoRI-F CCG GAA TTC ATG TTC CCG CCA TCA CCT TAC GGC GGA<br>P1427-Vps23A-BamHI-R GGG GGA TCC TCA TGA ATG TAA CCT ACC TGC GAT GGC TGC AA

MBP-Vps23A CC	Q1144-Vps23A(CC)-BamHI-F GGG GGA TCC ATG TTT GCT CGT GAT CCG CCT CTT TAT TCT CGA CG<br>Q1145-VPS23A(CC)-SalI-R GGG GTC GAC TCA ACA TTC AAA AGC ATT ATC CAC ATC CAA ATC AAC
MBP-Vps28A	Q1146-VPS28A-BamHI-F GGG GGA TCC ATG ATG GAG GTC AAA TTA TGG AAC<br>Q1147-VPS28A-SalI-R GGG GTC GAC TTA ATT ACC AGC TTT AGG CAA AGC
Cerulean-Vps28A	Q1146-VPS28A-SpeI-F GGG ACT AGT ATG ATG GAG GTC AAA TTA TGG AAC<br>Q1147-VPS28A-SacI-R GGG GAG CTC TTA ATT ACC AGC TTT AGG CAA AGC
Myc-SNF7A	O306-Myc-SNF7.1-SpeI-F GGG ACTAGT ATG ATG AAT CGG CTA TTC GGG AAA CCC AAG<br>O307-Myc-SNF7.1-XhoI-R GGG CTC GAG TTA GAG GGC CAT CTC AGC CTG AGC TGC AGC AGC TTC TTC TTC CTC AGC AGT CCG C
Myc-SNF7B	O308-Myc-SNF7.2-XbaI-F CGGTCTAGA ATG TTT ATG AAT CGG CTA TTC GGG AAA CCC<br>O309-Myc-SNF7.2-XhoI-R GGG CTC GAG TTA GAG AGC CAT CTC AGC TTG AGC GGC AGC AGC TTC ATC TTC CTC AGC AGT AGG TTG
pDONR-BRAFpro::BRAF(C3A)<br>pDONR-BRAFpro::BRAF(C3A,A330V)	O502-GW-pro-F<br>Q1234-P524-524(CA)-Bridge-R CAC AAC AGC AAT AGT AGA TTG AGC GGC CCC CAT GCT CTC TTT CTC TCT CTC TCT CTC TCT<br>Q1233-P524-524(CA) -Bridge-F AGA GAG AGA GAG AGA GAG AAA GAG AGC ATG GGG GCC GCT CAA TCT ACT ATT GCT GTT GTG<br>N1619-GW-R
DEX::SNF7.1(L32W)	SNF7-GW1-FF<br>GGGGACAAGTTTGTACAAAAAAGCAGGCTTGATGATGAATCGGCTATTCGGGAAAC<br>SNF7-GW219-6*HIS-RR<br>GGGGACCACTTTGTACAAGAAAGCTGGGTATCAATGATGATGATGATGATGGAGGGCCATCTCAGCCTGTAATGCAGC
DEX::Vps4(E232Q)	VPS4-GW1-FF<br>GGGGACAAGTTTGTACAAAAAAGCAGGCTTGATGTACAGCAATTTCAAGGAACAAG

	VPS4-GW435-6*HIS-RR<br>GGGGACCACTTTGTACAAGAAAGCTGGGTATCAATGATGATGAT GATGATGACCTTCTTCTCCAAACTCCTGTGTGAATC
braf-2 Genotyping	N1725 - SALK_145102-LP GCA ACT CAG AGA TGG CTG AAC<br>N1726 - SALK_145102-RP AGG GAA GTG TGT TTG CAC TTG<br>E98-T-DNA-LBa1 TGG TTC ACG TAG TGG GCC ATC G
braf-3 Genotyping	N1749 - 134H01-LP ATG TAC TAG AGT TCC ATG TCC TGC T<br>N1750 - 134H01-RP GAA TTA TTT TGT ATC TAT CAG GGC TCC<br>K588-o8474 ATA ATA ACG CTG CGG ACA TCT ACA TTT T
braf-1 Sanger sequencing	N1607-524-G989A-LP AAT GAT CTC ATT ATC ATT TCA GGC A<br>N1608-524-G989A-RP GGC AGT TCA AAG TCG TCG GGT CTC A
BRAF qRT-PCR	O1232-P1-F GTG AGC CGG CTT GTC ACT TGA GCG CT<br>O189-P2-R GGG GGT ACC TGA GGA AGA GAC ATC CTC TAT TTC AGG CAG TTC AAA
UBQ10 qRT-PCR	N491-UBQ10-RT1 GATCTTTGCCGGAAAACAATTGGAGGATGGT<br>N492-UBQ10-RT2 CGACTTGTCATTAGAAAGAAAGAGATAACAGG
FREE1-RNAi Genotyping	N1333-Fi-LP GCTTCTCGAAACCCATTTCATC<br>N1334-Fi-RP GAGCTTCAGGCTTCATAGGT<br>N1335-Fi-TP ATTTCGGAACCACCATCAAACAG
